# 13-Ethoxy­carbonyl-16-(1-methyl­ethyl)-17,19-dinoratis-15-ene-4,14-dicarboxylic acid monohydrate: a new derivative of maleopimaric acid

**DOI:** 10.1107/S1600536809028141

**Published:** 2009-07-22

**Authors:** Meng Zhang, Xiao-xin Guo, Yong-hong Zhou, Hong-jun Liu

**Affiliations:** a Institute of Chemical Industry of Forest Products, Chinese Academy of Forestry, Nanjing 210042, People’s Republic of China

## Abstract

The title compound, C_26_H_38_O_6_·H_2_O, is a mono-ester of a derivative of maleopimaric acid, an abietic-type acid. The two fused and unbridged cyclo­hexane rings adopt approximate chair conformations while the three other three six-membered rings have boat conformations.

## Related literature

Abietic type resin acid, the major component of gum rosin, is a high quality biomass resource for the development of new chiral drugs, see: McCoy (2000 ▶); Schweizer *et al.* (2003 ▶). For the use of abietic acid and its derivatives in the design and synthesis of industrially and physiologically important products, see: Savluchinske-Feio *et al.* (2007 ▶). For the structures of other maleopimaric acid derivatives, see: Li *et al.* (2005 ▶); Pan *et al.* (2006 ▶); Rao *et al.* (2006 ▶).
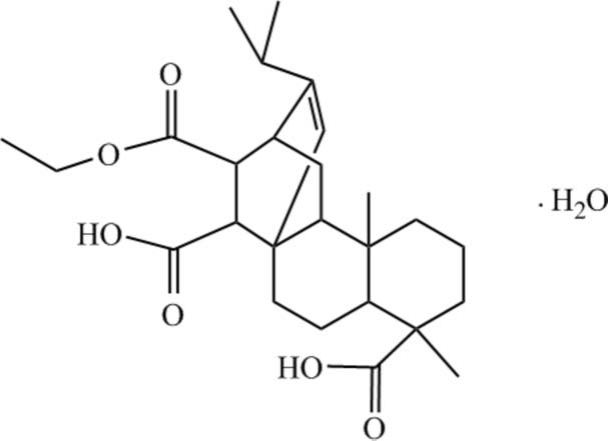


## Experimental

### 

#### Crystal data

C_26_H_38_O_6_·H_2_O
*M* *_r_* = 464.58
Orthorhombic, 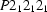

*a* = 7.3406 (14) Å
*b* = 17.901 (4) Å
*c* = 19.681 (4) Å
*V* = 2586.2 (9) Å^3^
*Z* = 4
Mo *K*α radiation
μ = 0.09 mm^−1^
*T* = 291 K
0.26 × 0.22 × 0.20 mm

#### Data collection

Bruker SMART APEX CCD diffractometer
Absorption correction: multi-scan (*SADABS*; Bruker, 2000 ▶) *T*_min_ = 0.98, *T*_max_ = 0.98
14084 measured reflections
2892 independent reflections
2318 reflections with *I* > 2σ(*I*)
*R*_int_ = 0.076

#### Refinement

*R*[*F* ^2^ > 2σ(*F* ^2^)] = 0.054
*wR*(*F* ^2^) = 0.113
*S* = 1.08
2892 reflections
303 parameters
H-atom parameters constrained
Δρ_max_ = 0.19 e Å^−3^
Δρ_min_ = −0.22 e Å^−3^

### 

Data collection: *SMART* (Bruker, 2000 ▶); cell refinement: *SAINT* (Bruker, 2000 ▶); data reduction: *SAINT*; program(s) used to solve structure: *SHELXS97* (Sheldrick, 2008 ▶); program(s) used to refine structure: *SHELXL97* (Sheldrick, 2008 ▶); molecular graphics: *SHELXTL* (Sheldrick, 2008 ▶); software used to prepare material for publication: *SHELXTL*.

## Supplementary Material

Crystal structure: contains datablocks I, global. DOI: 10.1107/S1600536809028141/bh2234sup1.cif

Structure factors: contains datablocks I. DOI: 10.1107/S1600536809028141/bh2234Isup2.hkl

Additional supplementary materials:  crystallographic information; 3D view; checkCIF report

## Figures and Tables

**Fig. 1.**
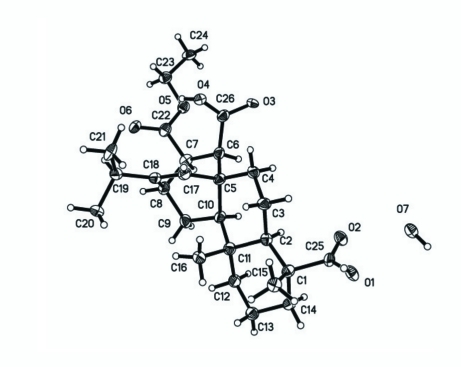
A view of the molecular structure of the title compound, showing displacement ellipsoids at the 30% probability level. Hydrogen atoms are drawn as spheres of arbitrary radius.

**Fig. 2.**
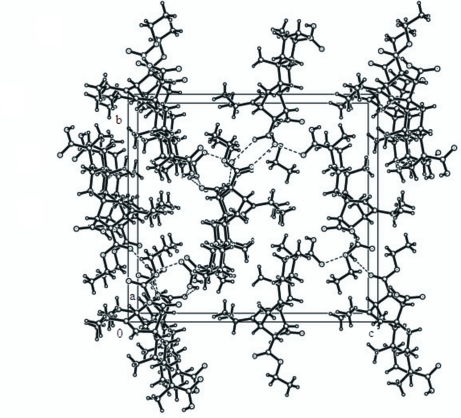
The packing of the title compound, viewed along the a axis. Dashed lines indicate hydrogen bonds.

## supplementary crystallographic information

### Comment

Rosin, a versatile natural resin, possesses a rare combination of many desirable properties and has consequently found innumerable industrial uses in a modified form or in conjunction with other natural or synthetic resins. Abietic type resin acid is the major component of gum rosin, including abietic acid, neoabietic acid, levo-pimaric acid, palustric acid, and is a high quality biomass resource in developing chiral new drug (McCoy, 2000; Schweizer *et al.*, 2003).

Abietic acid and its derivatives are readily available hydrophenanthrene compounds which become useful starting materials for the design and synthesis of industrially and physiologically important productions (Savluchinske-Feio *et al.*, 2007). The crystal structures of other derivatives of maleopimaric acid have already been published (Rao *et al.*, 2006; Li *et al.*, 2005; Pan *et al.*, 2006).

The molecular structure of the title compound is shown in Fig. 1. The asymmetric unit consists of two molecules, *viz*. maleopimaric ester derivative and one lattice water molecule. The cyclohexane rings C2···C5/C10/C11 and C1/C2/C11···C14 have typical chair forms. The cyclohexane ring C5···C10 has a slightly distorted boat conformation; the other two six-membered rings adopt boat conformations. The configuration about the C17C18 bond is *Z* (Fig. 1), with the H atom and the isopropyl group *cis* with respect to each other. The bond lengths and angles have normal values.

In the crystal structure, the molecules are linked (Fig. 2) by O—H···O and C—H···O intermolecular hydrogen bonds.

### Experimental

Abietic acid (30.2 g), acetic acid (20 ml), and maleic acid ethyl ester (14.4 g) were put into a 100-ml three-necked flask and magnetically stirred; the mixture was stirred for 25 min. The solution was then put into 5 ml of glacial acetic acid and cooled, washed with hot water (10 ml), dried (MgSO_4_), and concentrated to dryness. Recrystallization from ethanol (95%) afforded the adduct (33.9 g, 73%).

### Refinement

All H atoms were placed in geometrically idealized positions and constrained to ride on their parent atoms, with C—H distances in the range 0.93–0.98 Å, and O—H distances of 0.85 Å, with *U*_iso_(H) = 1.2 or 1.5*U*_eq_ of the carrier atom. In the absence of significant anomalous scattering effects, Friedel pairs were merged.

### Crystal data

**Table:**

C_26_H_38_O_6_·H_2_O	*D*_x_ = 1.193 Mg m^−^^3^
*M**_r_* = 464.58	Melting point: 412 K
Orthorhombic, *P*2_1_2_1_2_1_	Mo *K*α radiation, λ = 0.71073 Å
Hall symbol: P 2ac 2ab	Cell parameters from 2351 reflections
*a* = 7.3406 (14) Å	θ = 2.4–23.1°
*b* = 17.901 (4) Å	µ = 0.09 mm^−^^1^
*c* = 19.681 (4) Å	*T* = 291 K
*V* = 2586.2 (9) Å^3^	Aciculae, colorless
*Z* = 4	0.26 × 0.22 × 0.20 mm
*F*(000) = 1008	

### Data collection

**Table:**

Bruker SMART APEX CCD diffractometer	2892 independent reflections
Radiation source: sealed tube	2318 reflections with *I* > 2σ(*I*)
graphite	*R*_int_ = 0.076
φ and ω scans	θ_max_ = 26.0°, θ_min_ = 2.3°
Absorption correction: multi-scan (*SADABS*; Bruker, 2000)	*h* = −8→9
*T*_min_ = 0.98, *T*_max_ = 0.98	*k* = −21→22
14084 measured reflections	*l* = −11→24

### Refinement

**Table:**

Refinement on *F*^2^	Primary atom site location: structure-invariant direct methods
Least-squares matrix: full	Secondary atom site location: difference Fourier map
*R*[*F*^2^ > 2σ(*F*^2^)] = 0.054	Hydrogen site location: inferred from neighbouring sites
*wR*(*F*^2^) = 0.113	H-atom parameters constrained
*S* = 1.08	*w* = 1/[σ^2^(*F*_o_^2^) + (0.04*P*)^2^ + 0.66*P*] where *P* = (*F*_o_^2^ + 2*F*_c_^2^)/3
2892 reflections	(Δ/σ)_max_ < 0.001
303 parameters	Δρ_max_ = 0.19 e Å^−^^3^
0 restraints	Δρ_min_ = −0.22 e Å^−^^3^
0 constraints	

### Fractional atomic coordinates and isotropic or equivalent isotropic displacement parameters (Å^2^)

**Table:**

	*x*	*y*	*z*	*U*_iso_*/*U*_eq_	
C1	0.1062 (5)	0.27241 (19)	0.17771 (18)	0.0339 (8)	
C2	0.1205 (5)	0.18852 (19)	0.16317 (18)	0.0321 (8)	
H2	0.0801	0.1643	0.2052	0.039*	
C3	0.3180 (5)	0.1612 (2)	0.1530 (2)	0.0386 (9)	
H3A	0.3605	0.1755	0.1082	0.046*	
H3B	0.3968	0.1845	0.1864	0.046*	
C4	0.3272 (5)	0.0766 (2)	0.1604 (2)	0.0377 (9)	
H4A	0.3005	0.0636	0.2072	0.045*	
H4B	0.4505	0.0603	0.1507	0.045*	
C5	0.1958 (5)	0.03398 (19)	0.11385 (18)	0.0330 (8)	
C6	0.1724 (6)	−0.0481 (2)	0.14105 (18)	0.0378 (9)	
H6	0.1139	−0.0455	0.1858	0.045*	
C7	0.0419 (5)	−0.0908 (2)	0.0922 (2)	0.0382 (9)	
H7	−0.0692	−0.1011	0.1182	0.046*	
C8	−0.0146 (5)	−0.0396 (2)	0.03338 (19)	0.0406 (9)	
H8	−0.0907	−0.0669	0.0009	0.049*	
C9	−0.1211 (5)	0.0256 (2)	0.06435 (19)	0.0416 (9)	
H9A	−0.2277	0.0066	0.0878	0.050*	
H9B	−0.1621	0.0587	0.0284	0.050*	
C10	0.0003 (5)	0.07016 (19)	0.11538 (19)	0.0339 (8)	
H10	−0.0491	0.0597	0.1607	0.041*	
C11	−0.0061 (5)	0.1561 (2)	0.10698 (18)	0.0360 (8)	
C12	−0.2015 (5)	0.1820 (2)	0.1218 (2)	0.0411 (9)	
H12A	−0.2443	0.1576	0.1629	0.049*	
H12B	−0.2799	0.1665	0.0848	0.049*	
C13	−0.2173 (5)	0.2672 (2)	0.1309 (2)	0.0426 (9)	
H13A	−0.1819	0.2918	0.0891	0.051*	
H13B	−0.3430	0.2802	0.1404	0.051*	
C14	−0.0966 (5)	0.2947 (2)	0.1888 (2)	0.0394 (9)	
H14A	−0.1394	0.2737	0.2313	0.047*	
H14B	−0.1057	0.3487	0.1919	0.047*	
C15	0.1951 (6)	0.3238 (2)	0.1237 (2)	0.0407 (9)	
H15A	0.2085	0.3732	0.1420	0.061*	
H15B	0.1192	0.3256	0.0840	0.061*	
H15C	0.3127	0.3045	0.1117	0.061*	
C16	0.0475 (6)	0.1813 (2)	0.03444 (19)	0.0417 (9)	
H16A	−0.0103	0.1494	0.0017	0.063*	
H16B	0.1773	0.1782	0.0293	0.063*	
H16C	0.0087	0.2319	0.0274	0.063*	
C17	0.2593 (6)	0.0286 (2)	0.04130 (19)	0.0388 (9)	
H17	0.3666	0.0512	0.0268	0.047*	
C18	0.1540 (5)	−0.01020 (19)	−0.00112 (19)	0.0364 (8)	
C19	0.1871 (5)	−0.0250 (2)	−0.07525 (19)	0.0433 (10)	
H19	0.1618	−0.0780	−0.0832	0.052*	
C20	0.0537 (6)	0.0199 (2)	−0.1194 (2)	0.0452 (10)	
H20A	−0.0670	0.0156	−0.1010	0.068*	
H20B	0.0552	0.0007	−0.1649	0.068*	
H20C	0.0895	0.0715	−0.1197	0.068*	
C21	0.3860 (6)	−0.0108 (2)	−0.0965 (2)	0.0467 (10)	
H21A	0.4165	0.0406	−0.0883	0.070*	
H21B	0.4001	−0.0217	−0.1440	0.070*	
H21C	0.4654	−0.0424	−0.0706	0.070*	
C22	0.1092 (6)	−0.1652 (2)	0.0662 (2)	0.0415 (9)	
C23	0.2168 (6)	−0.2869 (2)	0.0970 (2)	0.0429 (9)	
H23A	0.1186	−0.3141	0.0752	0.051*	
H23B	0.3167	−0.2825	0.0650	0.051*	
C24	0.2779 (6)	−0.3275 (2)	0.1586 (2)	0.0447 (10)	
H24A	0.3775	−0.3011	0.1792	0.067*	
H24B	0.3169	−0.3769	0.1462	0.067*	
H24C	0.1789	−0.3309	0.1904	0.067*	
C25	0.1958 (5)	0.2900 (2)	0.24603 (19)	0.0371 (8)	
C26	0.3572 (5)	−0.0844 (2)	0.1500 (2)	0.0384 (9)	
O1	0.2639 (4)	0.35119 (14)	0.25908 (13)	0.0424 (7)	
H1B	0.3484	0.3602	0.2308	0.051*	
O2	0.1860 (4)	0.23935 (14)	0.29190 (13)	0.0423 (7)	
O3	0.4065 (4)	−0.09128 (14)	0.21215 (13)	0.0436 (7)	
O4	0.4538 (4)	−0.10191 (15)	0.10165 (13)	0.0409 (6)	
H4D	0.3978	−0.1341	0.0778	0.049*	
O5	0.1529 (4)	−0.21151 (13)	0.11749 (13)	0.0391 (6)	
O6	0.1159 (3)	−0.18329 (13)	0.00746 (14)	0.0408 (6)	
O7	0.2953 (4)	0.28560 (15)	0.40900 (13)	0.0425 (6)	
H7A	0.2924	0.3307	0.4224	0.051*	
H7B	0.2238	0.2594	0.4333	0.051*	

### Atomic displacement parameters (Å^2^)

**Table:**

	*U*^11^	*U*^22^	*U*^33^	*U*^12^	*U*^13^	*U*^23^
C1	0.0347 (19)	0.0271 (17)	0.040 (2)	−0.0036 (16)	0.0019 (17)	−0.0020 (15)
C2	0.0265 (18)	0.0361 (19)	0.0337 (19)	−0.0052 (15)	−0.0003 (16)	−0.0016 (16)
C3	0.0275 (19)	0.040 (2)	0.048 (2)	0.0030 (16)	−0.0046 (18)	−0.0039 (17)
C4	0.0325 (19)	0.037 (2)	0.044 (2)	0.0000 (16)	−0.0080 (17)	−0.0032 (17)
C5	0.0271 (18)	0.0347 (19)	0.037 (2)	0.0018 (14)	0.0040 (16)	−0.0036 (16)
C6	0.048 (2)	0.038 (2)	0.0274 (19)	0.0047 (18)	0.0051 (17)	−0.0015 (15)
C7	0.035 (2)	0.0338 (19)	0.045 (2)	−0.0025 (16)	0.0062 (17)	−0.0028 (18)
C8	0.043 (2)	0.042 (2)	0.037 (2)	0.0004 (17)	−0.0015 (19)	−0.0089 (18)
C9	0.036 (2)	0.051 (2)	0.037 (2)	−0.0026 (18)	−0.0058 (18)	−0.0076 (18)
C10	0.0252 (17)	0.039 (2)	0.038 (2)	0.0000 (15)	0.0017 (17)	−0.0099 (16)
C11	0.0354 (19)	0.0377 (19)	0.035 (2)	−0.0004 (16)	0.0016 (18)	−0.0008 (16)
C12	0.0298 (19)	0.049 (2)	0.044 (2)	0.0025 (17)	−0.0049 (17)	−0.0099 (19)
C13	0.035 (2)	0.045 (2)	0.048 (2)	0.0020 (17)	−0.0037 (18)	−0.0020 (18)
C14	0.040 (2)	0.037 (2)	0.042 (2)	0.0057 (17)	0.0020 (18)	−0.0030 (17)
C15	0.041 (2)	0.039 (2)	0.042 (2)	−0.0034 (17)	0.0019 (18)	0.0011 (17)
C16	0.048 (2)	0.039 (2)	0.039 (2)	0.0049 (18)	−0.0066 (18)	0.0010 (17)
C17	0.041 (2)	0.036 (2)	0.040 (2)	0.0054 (17)	0.0039 (18)	−0.0010 (16)
C18	0.040 (2)	0.0335 (19)	0.0356 (19)	0.0076 (16)	−0.0004 (17)	0.0017 (17)
C19	0.042 (2)	0.053 (2)	0.035 (2)	0.0143 (19)	0.0014 (18)	0.0017 (18)
C20	0.047 (2)	0.046 (2)	0.043 (2)	0.0144 (18)	−0.0070 (19)	0.0063 (18)
C21	0.054 (3)	0.045 (2)	0.041 (2)	−0.0068 (19)	0.021 (2)	−0.0155 (19)
C22	0.048 (2)	0.032 (2)	0.044 (2)	−0.0064 (18)	0.005 (2)	−0.0064 (17)
C23	0.038 (2)	0.047 (2)	0.044 (2)	0.0031 (18)	0.0079 (19)	−0.0003 (19)
C24	0.042 (2)	0.046 (2)	0.046 (2)	0.0153 (19)	0.0146 (19)	0.0098 (18)
C25	0.038 (2)	0.037 (2)	0.0358 (19)	−0.0016 (17)	−0.0063 (17)	−0.0104 (17)
C26	0.045 (2)	0.0295 (19)	0.041 (2)	0.0031 (17)	0.0013 (18)	−0.0051 (16)
O1	0.0412 (15)	0.0444 (15)	0.0415 (15)	−0.0124 (13)	−0.0165 (13)	0.0077 (12)
O2	0.0409 (15)	0.0438 (15)	0.0423 (14)	−0.0094 (12)	−0.0090 (13)	0.0049 (12)
O3	0.0449 (16)	0.0410 (14)	0.0449 (16)	0.0214 (13)	−0.0168 (13)	−0.0026 (12)
O4	0.0414 (15)	0.0439 (16)	0.0375 (15)	0.0136 (12)	−0.0009 (12)	−0.0033 (12)
O5	0.0464 (15)	0.0319 (13)	0.0391 (14)	−0.0007 (12)	0.0035 (12)	−0.0011 (11)
O6	0.0421 (15)	0.0375 (14)	0.0429 (15)	0.0016 (12)	0.0042 (13)	−0.0123 (12)
O7	0.0413 (15)	0.0449 (15)	0.0413 (15)	−0.0066 (12)	−0.0096 (12)	0.0059 (13)

### Geometric parameters (Å, °)

**Table:**

C1—C25	1.530 (5)	C14—H14A	0.9700
C1—C2	1.532 (5)	C14—H14B	0.9700
C1—C15	1.550 (5)	C15—H15A	0.9600
C1—C14	1.557 (5)	C15—H15B	0.9600
C2—C3	1.543 (5)	C15—H15C	0.9600
C2—C11	1.557 (5)	C16—H16A	0.9600
C2—H2	0.9800	C16—H16B	0.9600
C3—C4	1.523 (5)	C16—H16C	0.9600
C3—H3A	0.9700	C17—C18	1.333 (5)
C3—H3B	0.9700	C17—H17	0.9300
C4—C5	1.533 (5)	C18—C19	1.503 (5)
C4—H4A	0.9700	C19—C20	1.535 (5)
C4—H4B	0.9700	C19—C21	1.540 (6)
C5—C17	1.505 (5)	C19—H19	0.9800
C5—C6	1.574 (5)	C20—H20A	0.9600
C5—C10	1.574 (5)	C20—H20B	0.9600
C6—C26	1.514 (5)	C20—H20C	0.9600
C6—C7	1.557 (5)	C21—H21A	0.9600
C6—H6	0.9800	C21—H21B	0.9600
C7—C22	1.510 (5)	C21—H21C	0.9600
C7—C8	1.534 (5)	C22—O6	1.201 (4)
C7—H7	0.9800	C22—O5	1.345 (5)
C8—C18	1.506 (5)	C23—C24	1.484 (5)
C8—C9	1.532 (5)	C23—O5	1.484 (5)
C8—H8	0.9800	C23—H23A	0.9700
C9—C10	1.562 (5)	C23—H23B	0.9700
C9—H9A	0.9700	C24—H24A	0.9600
C9—H9B	0.9700	C24—H24B	0.9600
C10—C11	1.548 (5)	C24—H24C	0.9600
C10—H10	0.9800	C25—O1	1.231 (4)
C11—C12	1.535 (5)	C25—O2	1.281 (4)
C11—C16	1.548 (5)	C26—O4	1.227 (4)
C12—C13	1.539 (5)	C26—O3	1.282 (4)
C12—H12A	0.9700	O1—H1B	0.8499
C12—H12B	0.9700	O4—H4D	0.8500
C13—C14	1.524 (5)	O7—H7A	0.8499
C13—H13A	0.9700	O7—H7B	0.8501
C13—H13B	0.9700		
			
C25—C1—C2	109.6 (3)	C14—C13—H13A	109.4
C25—C1—C15	107.4 (3)	C12—C13—H13A	109.4
C2—C1—C15	115.1 (3)	C14—C13—H13B	109.4
C25—C1—C14	103.6 (3)	C12—C13—H13B	109.4
C2—C1—C14	110.1 (3)	H13A—C13—H13B	108.0
C15—C1—C14	110.2 (3)	C13—C14—C1	111.6 (3)
C1—C2—C3	113.6 (3)	C13—C14—H14A	109.3
C1—C2—C11	117.2 (3)	C1—C14—H14A	109.3
C3—C2—C11	110.5 (3)	C13—C14—H14B	109.3
C1—C2—H2	104.7	C1—C14—H14B	109.3
C3—C2—H2	104.7	H14A—C14—H14B	108.0
C11—C2—H2	104.7	C1—C15—H15A	109.5
C4—C3—C2	110.1 (3)	C1—C15—H15B	109.5
C4—C3—H3A	109.6	H15A—C15—H15B	109.5
C2—C3—H3A	109.6	C1—C15—H15C	109.5
C4—C3—H3B	109.6	H15A—C15—H15C	109.5
C2—C3—H3B	109.6	H15B—C15—H15C	109.5
H3A—C3—H3B	108.1	C11—C16—H16A	109.5
C3—C4—C5	114.2 (3)	C11—C16—H16B	109.5
C3—C4—H4A	108.7	H16A—C16—H16B	109.5
C5—C4—H4A	108.7	C11—C16—H16C	109.5
C3—C4—H4B	108.7	H16A—C16—H16C	109.5
C5—C4—H4B	108.7	H16B—C16—H16C	109.5
H4A—C4—H4B	107.6	C18—C17—C5	116.6 (4)
C17—C5—C4	113.8 (3)	C18—C17—H17	121.7
C17—C5—C6	107.3 (3)	C5—C17—H17	121.7
C4—C5—C6	109.3 (3)	C17—C18—C19	127.3 (4)
C17—C5—C10	109.1 (3)	C17—C18—C8	112.1 (3)
C4—C5—C10	110.9 (3)	C19—C18—C8	120.6 (3)
C6—C5—C10	106.2 (3)	C18—C19—C20	110.7 (3)
C26—C6—C7	114.4 (3)	C18—C19—C21	112.9 (3)
C26—C6—C5	110.0 (3)	C20—C19—C21	111.4 (4)
C7—C6—C5	108.4 (3)	C18—C19—H19	107.2
C26—C6—H6	108.0	C20—C19—H19	107.2
C7—C6—H6	108.0	C21—C19—H19	107.2
C5—C6—H6	108.0	C19—C20—H20A	109.5
C22—C7—C8	111.1 (3)	C19—C20—H20B	109.5
C22—C7—C6	116.1 (3)	H20A—C20—H20B	109.5
C8—C7—C6	109.8 (3)	C19—C20—H20C	109.5
C22—C7—H7	106.4	H20A—C20—H20C	109.5
C8—C7—H7	106.4	H20B—C20—H20C	109.5
C6—C7—H7	106.4	C19—C21—H21A	109.5
C18—C8—C9	109.4 (3)	C19—C21—H21B	109.5
C18—C8—C7	109.1 (3)	H21A—C21—H21B	109.5
C9—C8—C7	107.0 (3)	C19—C21—H21C	109.5
C18—C8—H8	110.4	H21A—C21—H21C	109.5
C9—C8—H8	110.4	H21B—C21—H21C	109.5
C7—C8—H8	110.4	O6—C22—O5	123.1 (3)
C8—C9—C10	110.7 (3)	O6—C22—C7	125.3 (4)
C8—C9—H9A	109.5	O5—C22—C7	111.5 (3)
C10—C9—H9A	109.5	C24—C23—O5	108.6 (3)
C8—C9—H9B	109.5	C24—C23—H23A	110.0
C10—C9—H9B	109.5	O5—C23—H23A	110.0
H9A—C9—H9B	108.1	C24—C23—H23B	110.0
C11—C10—C9	114.9 (3)	O5—C23—H23B	110.0
C11—C10—C5	115.8 (3)	H23A—C23—H23B	108.3
C9—C10—C5	107.3 (3)	C23—C24—H24A	109.5
C11—C10—H10	106.0	C23—C24—H24B	109.5
C9—C10—H10	106.0	H24A—C24—H24B	109.5
C5—C10—H10	106.0	C23—C24—H24C	109.5
C12—C11—C16	109.0 (3)	H24A—C24—H24C	109.5
C12—C11—C10	107.9 (3)	H24B—C24—H24C	109.5
C16—C11—C10	112.3 (3)	O1—C25—O2	120.3 (3)
C12—C11—C2	108.1 (3)	O1—C25—C1	122.7 (3)
C16—C11—C2	113.3 (3)	O2—C25—C1	116.7 (3)
C10—C11—C2	106.0 (3)	O4—C26—O3	123.5 (4)
C11—C12—C13	113.0 (3)	O4—C26—C6	122.5 (4)
C11—C12—H12A	109.0	O3—C26—C6	113.9 (3)
C13—C12—H12A	109.0	C25—O1—H1B	109.1
C11—C12—H12B	109.0	C26—O4—H4D	108.8
C13—C12—H12B	109.0	C22—O5—C23	115.6 (3)
H12A—C12—H12B	107.8	H7A—O7—H7B	109.5
C14—C13—C12	111.3 (3)		
			
C25—C1—C2—C3	−65.4 (4)	C3—C2—C11—C12	177.2 (3)
C15—C1—C2—C3	55.8 (4)	C1—C2—C11—C16	70.3 (4)
C14—C1—C2—C3	−178.8 (3)	C3—C2—C11—C16	−61.9 (4)
C25—C1—C2—C11	163.8 (3)	C1—C2—C11—C10	−166.1 (3)
C15—C1—C2—C11	−75.0 (4)	C3—C2—C11—C10	61.7 (4)
C14—C1—C2—C11	50.4 (4)	C16—C11—C12—C13	−70.6 (4)
C1—C2—C3—C4	162.8 (3)	C10—C11—C12—C13	167.2 (3)
C11—C2—C3—C4	−63.2 (4)	C2—C11—C12—C13	52.9 (4)
C2—C3—C4—C5	54.5 (4)	C11—C12—C13—C14	−58.7 (4)
C3—C4—C5—C17	77.7 (4)	C12—C13—C14—C1	56.6 (4)
C3—C4—C5—C6	−162.5 (3)	C25—C1—C14—C13	−168.7 (3)
C3—C4—C5—C10	−45.8 (4)	C2—C1—C14—C13	−51.5 (4)
C17—C5—C6—C26	70.7 (4)	C15—C1—C14—C13	76.6 (4)
C4—C5—C6—C26	−53.1 (4)	C4—C5—C17—C18	177.6 (3)
C10—C5—C6—C26	−172.8 (3)	C6—C5—C17—C18	56.6 (4)
C17—C5—C6—C7	−55.0 (4)	C10—C5—C17—C18	−57.9 (4)
C4—C5—C6—C7	−178.8 (3)	C5—C17—C18—C19	−179.1 (3)
C10—C5—C6—C7	61.5 (4)	C5—C17—C18—C8	1.6 (5)
C26—C6—C7—C22	5.7 (5)	C9—C8—C18—C17	57.1 (4)
C5—C6—C7—C22	128.8 (3)	C7—C8—C18—C17	−59.7 (4)
C26—C6—C7—C8	−121.4 (3)	C9—C8—C18—C19	−122.3 (4)
C5—C6—C7—C8	1.8 (4)	C7—C8—C18—C19	120.9 (3)
C22—C7—C8—C18	−74.8 (4)	C17—C18—C19—C20	−108.6 (4)
C6—C7—C8—C18	55.0 (4)	C8—C18—C19—C20	70.7 (5)
C22—C7—C8—C9	166.9 (3)	C17—C18—C19—C21	17.0 (6)
C6—C7—C8—C9	−63.3 (4)	C8—C18—C19—C21	−163.7 (3)
C18—C8—C9—C10	−57.4 (4)	C8—C7—C22—O6	−1.2 (6)
C7—C8—C9—C10	60.7 (4)	C6—C7—C22—O6	−127.6 (4)
C8—C9—C10—C11	133.3 (3)	C8—C7—C22—O5	−177.8 (3)
C8—C9—C10—C5	3.0 (4)	C6—C7—C22—O5	55.8 (4)
C17—C5—C10—C11	−78.5 (4)	C2—C1—C25—O1	152.3 (4)
C4—C5—C10—C11	47.7 (4)	C15—C1—C25—O1	26.5 (5)
C6—C5—C10—C11	166.3 (3)	C14—C1—C25—O1	−90.2 (4)
C17—C5—C10—C9	51.4 (4)	C2—C1—C25—O2	−32.6 (4)
C4—C5—C10—C9	177.5 (3)	C15—C1—C25—O2	−158.4 (3)
C6—C5—C10—C9	−63.9 (4)	C14—C1—C25—O2	84.9 (4)
C9—C10—C11—C12	63.4 (4)	C7—C6—C26—O4	52.3 (5)
C5—C10—C11—C12	−170.5 (3)	C5—C6—C26—O4	−69.9 (4)
C9—C10—C11—C16	−56.8 (4)	C7—C6—C26—O3	−131.2 (3)
C5—C10—C11—C16	69.3 (4)	C5—C6—C26—O3	106.5 (4)
C9—C10—C11—C2	179.0 (3)	O6—C22—O5—C23	2.0 (5)
C5—C10—C11—C2	−54.9 (4)	C7—C22—O5—C23	178.7 (3)
C1—C2—C11—C12	−50.6 (4)	C24—C23—O5—C22	173.9 (3)

### Hydrogen-bond geometry (Å, °)

**Table:**

*D*—H···*A*	*D*—H	H···*A*	*D*···*A*	*D*—H···*A*
O4—H4D···O5	0.85	2.40	2.971 (4)	125
O7—H7B···O6^i^	0.85	2.32	2.745 (4)	111
C2—H2···O2	0.98	2.31	2.735 (4)	105
C15—H15A···O1	0.96	2.37	2.756 (5)	103

Symmetry codes: (i) −*x*+1/2, −*y*, *z*+1/2.
